# Supplementary material for: Unraveling the impact of congenital deafness on individual brain organization
Source: eLife. 2025 Mar 12;13:RP96944. doi: 10.7554/eLife.96944 (PMC11903032; doi:10.7554/eLife.96944)
Supplement: Supplementary file 1. [file elife-96944-supp1.docx]

| **Supplementary Table 1.** MNI coordinates (x, y, z) for the FC variability analyses. | | | | | |
| --- | --- | --- | --- | --- | --- |
|  |  | **Deaf vs. Hearing (peak F-value) Fig. 1A** | **Deaf Native vs. Hearing (peak F-value) Fig. 1C** | **Deaf Delayed vs. Hearing (peak F-value) Fig. 1–figure supplement 1** | **Deaf Delayed vs. Deaf Native (peak F-value) Fig. 3A** |
| **Left Hemisphere** | *Superior Temporal Gyrus* | -56 -16 4 (15) | -64 -28 0 (15.7) |  |  |
|  | *Middle Temporal Gyrus* | -64 -44 0 (16.3) | -66 -46 2 (17.2) |  |  |
|  | *Inferior Frontal Gyrus* | -40 20 24 (15.5) | -46 28 20 (30.4) | -40 20 24 (13.2) |  |
|  | *Middle Frontal Gyrus* |  | -36 30 36 (19.3) |  |  |
|  | *Posterior Middle Frontal Gyrus* |  |  |  | -54 -4 48 (13.7) |
|  | *Visual V3* | -20 -100 14 (18.1) |  | -14 -94 20 (15.7) |  |
|  | *Lateral Occipital Cortex (dorsal stream)* | -16 -66 50 (17.5) |  | -16 -66 50 (14.7) |  |
|  | *Supramarginal Gyrus* |  |  |  | -50 -42 34 (21.1) |
|  | *Precuneus* |  |  |  | -18 -64 26 (12) |
|  | *Inferior Parietal Lobe* | -38 -84 32 (11.9) |  |  |  |
|  | *Precentral Gyrus* | -2 -26 72 (10.9) | -4 -26 66 (10.9) |  |  |
|  | *Pre-Supplementary Motor Area* | -2 8 56 (14.9) |  | -2 6 56 (18.2) |  |
| **Right Hemisphere** | *Postcentral Gyrus* | 12 -42 70 (21.6) |  |  |  |
|  | *Precentral Gyrus* |  | 4 -14 56 (23.4) |  |  |
|  | *Pre-Supplementary Motor Area* | 4 10 60 (20.9) |  | 4 12 58 (31.9) |  |
|  | *Inferior Frontal Gyrus* | 46 22 24 (27.6) | 44 24 24 (20.2) | 44 20 26 (29.1) |  |
|  | *Anterior Inferior Frontal Gyrus* |  |  | 42 28 8 (14.6) | 44 34 -8 (16) |
|  | *Middle Frontal Gyrus* |  |  |  |  |
|  | *Frontal pole* | 48 48 12 (17) | 46 48 12 (19.3) |  |  |
|  | *Supramarginal gyrus* |  | 66 -38 40 (14.9) |  |  |
|  | *Superior Parietal Lobe* |  | 6 -76 48 (13.7) |  |  |
