# Supplementary material for: Unraveling the impact of congenital deafness on individual brain organization
Source: eLife. 2025 Mar 12;13:RP96944. doi: 10.7554/eLife.96944 (PMC11903032; doi:10.7554/eLife.96944)
Supplement: Supplementary file 2. [file elife-96944-supp2.docx]

| **Supplementary Table 2.** Additional characteristics of deaf participants. | | | | | | | | | | |
| --- | --- | --- | --- | --- | --- | --- | --- | --- | --- | --- |
| **Sub no.** | **Age** | **Sex** | **Years of education** | **Age of deafness (months)** | **Hearing loss-left ear (dB)** | **Hearing loss-right ear (dB)** | **Age of sign language acquisition** | **Hearing aid use** | **Age of hearing aid** | **Hearing aid use duration (years)** |
| 34 | 22 | female | 15 | 0 | 120 | 110 | 7 | used in the past | 8 | 14 |
| 35 | 27 | male | 16 | 0 | 120 | 100 | 5 | uses currently | 3 | 24 |
| 36 | 22 | female | 14 | 0 | 110 | 110 | 8 | used in the past | 8 | 1 |
| 37 | 23 | female | 14 | 0 | 71-90 | 71-90 | 8 | used in the past | 15 | 0.5 |
| 38 | 22 | male | 12 | 0 | 120 | 120 | 8 | never used | - | 0 |
| 39 | 22 | male | 12 | 0 | >90 | >90 | 10 | never used | - | 0 |
| 40 | 23 | male | 15 | 0 | 71-90 | 71-90 | 9 | used in the past | 15 | 1 |
| 41 | 24 | male | 16 | 0 | >90 | >90 | 7 | never used | - | 0 |
| 42 | 23 | female | 15 | 0 | 71-90 | 71-90 | 4 | uses currently | 12 | 11 |
| 43 | 22 | female | 14 | 0 | 71-90 | 71-90 | 6 | used in the past | 8 | 9 |
| 44 | 24 | female | 16 | 0 | 105 | 105 | 7 | uses currently | 2 | 22 |
| 45 | 20 | female | 12 | 0 | >90 | >90 | 6 | used in the past | 6 | 14 |
| 46 | 24 | male | 16 | 0 | 95 | 90 | 5 | used in the past | 6 | 8 |
| 47 | 31 | male | 16 | 12 | 99 | 100 | 8 | never used | - | 0 |
| 48 | 42 | male | 16 | 6 | 93 | 93 | 7 | used in the past | 8 | 3 |
| 49 | 39 | male | 16 | 0 | 100 | 90 | 5 | used sporadically | 10 | Unknown |
| 50 | 31 | female | 16 | 0 | >90 | >90 | 5 | used in the past | 5 | 20 |
| 51 | 34 | female | 16 | 0 | >90 | 71-90 | 5 | used in the past | 12 | 10 |
| 52 | 33 | male | 16 | 0 | 100 | 90 | 9 | used in the past | 3 | 5 |
| 53 | 29 | female | 16 | 3 | 110 | 110 | 6 | uses currently | 25 | 4 |
| 54 | 30 | male | 16 | 0 | >90 | >90 | 8 | never used | - | 0 |
| 55 | 30 | male | 16 | 0 | 110 | 110 | 7 | used in the past | 3 | 5 |
| 56 | 26 | female | 16 | 0 | 71-90 | >90 | 9 | used in the past | 9 | 3 |
| 57 | 23 | male | 14 | 0 | 71-90 | >90 | 0 | never used | - | 0 |
| 58 | 21 | male | 13 | 0 | 103 | 105 | 0 | used in the past | 3 | 10 |
| 59 | 23 | male | 15 | 0 | 120 | 120 | 0 | never used | - | 0 |
| 60 | 23 | male | 15 | 0 | 120 | 120 | 0 | used in the past | 1 | 4 |
| 61 | 23 | male | 14 | 0 | >90 | >90 | 0 | used in the past | 8 | 8 |
| 62 | 28 | female | 15 | 0 | >90 | >90 | 0 | never used | - | 0 |
| 63 | 39 | female | 16 | 0 | 90 | 110 | 0 | never used | - | 0 |
| 64 | 21 | male | 12 | 0 | 90 | 91 | 0 | never used | - | 0 |
| 65 | 37 | female | 15 | 36 | 85 | 92 | 0 | used in the past | 6 | 1 |
| 66 | 22 | male | 9 | 0 | 120 | 120 | 0 | used in the past | 8 | 2 |
| 67 | 34 | male | 16 | 0 | 100 | 100 | 0 | uses currently | 3 | 31 |
| 68 | 30 | female | 16 | 0 | 110 | 110 | 0 | used in the past | 5 | 4 |
| 69 | 44 | male | 9 | 0 | >90 | >90 | 0 | used in the past | 30 | 0.5 |
| 70 | 25 | male | 16 | 0 | 90 | 90 | 0 | used in the past | 4 | 0.5 |
| 71 | 32 | male | 16 | 0 | 110 | 110 | 0 | never used | - | 0 |
| 72 | 31 | female | 15 | 0 | >90 | >90 | 0 | used in the past | 12 | 3 |
